# H Antigen expression modulates epidermal Keratinocyte Integrity and differentiation

**DOI:** 10.1186/s40659-024-00541-x

**Published:** 2024-10-18

**Authors:** Seon-Pil Jin, Jang-Hee Oh, Namjoo Kaylee Kim, Jin Ho Chung

**Affiliations:** 1 Department of Dermatology, Seoul National University Hospital, Seoul, Republic of Korea; 2 Institute of Human-Environment Interface Biology, Medical Research Center, Seoul National University, Seoul, Republic of Korea; 3 Department of Dermatology, Seoul National University College of Medicine, Seoul, Republic of Korea

**Keywords:** Blood group antigen, fucosyltransferase1, FUT1, Cell-cell adhesion, Desmosome, Glycosylation, Desmoglein, Epidermal growth factor receptor, EGFR

## Abstract

**Background:**

ABO blood group antigens (ABH antigens) are carbohydrate chains glycosylated on epithelial and red blood cells. Recent findings suggest reduced ABH expression in psoriasis and atopic dermatitis, a chronic inflammatory skin disease with retained scale. H antigen, a precursor for A and B antigens, is synthesized by fucosyltransferase 1 (FUT1). Desmosomes, critical for skin integrity, are known to require N-glycosylation for stability. We investigate the impact of H antigens, a specific type of glycosylation, on desmosomes in keratinocytes.

**Method:**

Primary human keratinocytes were transfected with FUT1 siRNA or recombinant adenovirus for FUT1 overexpression. Cell adhesion and desmosome characteristics and their underlying mechanisms were analyzed.

**Result:**

The knockdown of FUT1, responsible for H2 antigen expression in the skin, increased cell-cell adhesive strength and desmosome size in primary cultured keratinocytes without altering the overall desmosome structure. Desmosomal proteins, including desmogleins or plakophilin, were upregulated, suggesting enhanced desmosome assembly. Reduced H2 antigen expression via FUT1 knockdown led to increased keratinocyte differentiation, evidenced by elevated expression of differentiation markers. Epidermal growth factor receptor (EGFR) has been described to be associated with FUT1 and promotes cell migration and differentiation. The effects of FUT1 knockdown were recapitulated by an EGFR inhibitor concerning desmosomal proteins and cellular differentiation. Further investigation demonstrated that the FUT1 knockdown reduced EGFR signaling by lowering the levels of EGF ligands rather than directly regulating EGFR activity. Moreover, FUT1 overexpression reversed the effects observed in FUT1 knockdown, resulting in the downregulation of desmosomal proteins and differentiation markers while increasing both mRNA and protein levels of EGFR ligands.

**Conclusion:**

The expression level of FUT1 in the epidermis appears to influence cell-cell adhesion and keratinocyte differentiation status, at least partly through regulation of H2 antigen and EGFR ligand expression. These observations imply that the fucosylation of the H2 antigen by FUT1 could play a significant role in maintaining the molecular composition and regulation of desmosomes and suggest a possible involvement of the altered H2 antigen expression in skin diseases, such as psoriasis and atopic dermatitis.

**Supplementary Information:**

The online version contains supplementary material available at 10.1186/s40659-024-00541-x.

## Introduction

Cell-cell adhesion plays a key role in individual cells being organized into a multicellular organ. Skin epidermal keratinocytes have several different junctions: gap junctions, tight junctions, adherens junctions, and desmosomes [1]. Desmosomes are important to maintain skin integrity, emphasized by skin blistering disorder. Pemphigus and staphylococcal scalded skin syndrome have dysfunctional desmosomes by autoantibodies or bacterial toxins, respectively, which induce loss of cell-cell adhesion even with other kinds of intact junctions [2]. The ABO blood group antigens (ABH antigens) are carbohydrate chains attached to certain proteins or lipids, a part of glycosylation. In the human epidermis, fucosyltransferase 1 (FUT1) synthesizes H antigen by the addition of fucose onto precursor carbohydrates through an α-(1,2) linkage [α(1,2)-fucosylation]. Then, blood group A or B antigen is subsequently synthesized by A or B transferase [3]. Immunostaining analysis demonstrates that healthy skin with blood type O expresses both H2 antigen and FUT1 from the upper spinous layer to the horny layer of the epidermis [3, 4]. However, few reports have investigated the role of blood group antigens in the skin [5, 6]. Previously, we reported that N-glycosylation is critical to the stability of the desmosome complex [7]. Since FUT1 is an enzyme that affects the glycosylation status of the proteins, we initially studied if the H2 antigen also regulates desmosomes in primary human keratinocytes by *FUT1* knockdown siRNA transfection.

## Materials and methods

### Cell culture

Human primary epidermal keratinocytes were derived from foreskin tissue as described previously [8] and cultured in a keratinocyte growth medium (calcium concentration 0.1 mM) (KGM, KBM™ Gold™ Basal Medium with KGM™ Gold™ SingleQuots™ supplements, Lonza Bioscience, Durham, NC, USA). The cells were cultured in a humidified incubator in 5% CO2 at 37 °C. From second to fourth passages of keratinocytes were used for all experiments. Human tissue for primary cultures was obtained with the written informed consent of donors, under the approved protocol by the Institutional Review Board of Seoul National University Hospital. For siRNA transfection, cells were seeded on 35 mm culture dishes or 6 well plates and grown until > 90% confluence. To validate siRNA working, cells were harvested after 48 h. Except for siRNA validation experiments, after 24 h of siRNA transfection, the culture medium was switched to a fresh high calcium (2mM)-containing KGM, and the cells were incubated for indicated times. If the incubation time is longer than 2 days, the culture medium was replaced with a fresh high calcium-containing KGM at 48 h after the last medium switching. For the experiment of investigation of EGFR phosphorylation, after 24 h of siRNA transfection, cells were incubated in fresh KBM for 24 h, and then treated with 0 or 20 ng/ml of EGF in fresh KBM. Cells were harvested at 5 min after treatment with EGF. For an epidermal growth factor receptor (EGFR) inhibitor, gefitinib (Santa Cruz Biotechnologies, Santa Cruz, CA) treatment experiments, cells were grown until > 90% confluence, and then incubated with indicated doses of gefitinib (0, 20, or 100 nM) and high calcium-containing KGM for 4 days. The culture medium was additionally replaced with indicated doses of gefitinib and high calcium-containing fresh KGM at 48 h after incubation.

### siRNA transfection

Non-targeted negative control siRNA was used as a control siRNA (5’ – CCU ACG CCA CCA AUU UGC U – 3’(dTdT)). Two pools of *FUT1* siRNA were used to knockdown in this study. One pool (designated as #1) was purchased from Bioneer (Bioneer Corp., Daejeon, Korea), and composed of three different sequences of siRNAs (5’-CCG UGC UCA UUG CUA ACC A-3’(dTdT), (5’-CGA AAA GCG GAC UGU GGA U-3’(dTdT), and (5’-UGG UAC AGC UUC UGG AGC A-3’(dTdT)). The other (designated as #2) was SMARTPool ON-TARGETplus Human *FUT1* siRNA (2523) from Horizon Discovery Ltd. (Dharmacon™, Cambridge, United Kingdom), which is provided as a mixture of four different sequences of siRNAs. siRNA was transfected to keratinocytes at a confluence of > 90% using G-fectin (Genolution, Seoul, South Korea) according to the manufacturer’s manuals. Briefly, 400 nM of each siRNA pool was transfected using 4 µl of G-fectin transfection reagent per 35 mm dish. After 24 h, the culture medium was freshly changed. To confirm the specific downregulation of *FUT1* mRNA expression, control or FUT1 siRNA-transfected keratinocytes were harvested 2 days after the transfection, and the expressions of FUT1 mRNA were measured by real-time quantitative PCR, normalized to *36B4* mRNA expression.

### Western blot analysis

For Western blot analysis, at indicated times, cells were lysed with a lysis buffer (2% SDS, 2-mercaptoethanol (715 mM), 5% glycerol, 50 mM Tris pH 6.8, and 0.01% bromophenol blue) containing protease inhibitor cocktail (cOmplete™, Mini Protease Inhibitor Tablets, Roche, Mannheim, Germany) and phosphatase inhibitor cocktail 2 (Sigma-Aldrich, St. Louis, MO), and then boiled at 95ºC for 7 min. Same amounts of protein samples were separated by SDS-PAGE gel (6, 8, or 12%) and transferred onto nitrocellulose membranes. After blocking with 5% skim milk in Tris-buffered saline containing 0.1% Tween 20, membranes were incubated with antibodies specific for desmoglein 1 (B-11), desmoglein 3 (5H10), H type 2 antigen (BRIC231), α-tubulin (B-5-1-2), EGFR (A-10), filaggrin (AKH-1) (Santa Cruz Biotechnologies), p-EGFR(Y1068) (polyclonal, Cell Signaling Technology, Danvers, MA, USA), desmoplakin 1,2 (DP2.15, abcam, Cambridge, UK), desmocollin 1 (772906, R&D systems, Minneapolis, MN, USA), loricrin (Poly19051, Covance, Princeton, NJ, USA), plakophilin 1 (10B2) (Thermo Fisher Scientific, Waltham, MA), or FUT1 (rabbit polyclonal) (Cusabio Technology LLC, Houston, TX, USA). Membranes were washed using Tris-buffered saline with 0.1% Tween-20, incubated with secondary antibodies, and signals were detected using an enhanced chemiluminescent solution (Supersignal West Pico Plus or Femto, Thermo Scientific) with Amersham Imager 680 (GE Healthcare, Chicago, IL, USA). The densitometry of bands was analyzed using the Image J software (National Institutes of Health, Bethesda, MD, USA) and normalized with the quantified values of α-tubulin or β-actin as a loading control.

### Dispase-based dissociation assay

The cell-cell adhesive strength was assessed by dispase-based dissociation assay. After 48 h of siRNA transfection, when cells got a confluent cell monolayer, the culture medium was switched to a high calcium medium for an additional 8 h. The cells were then incubated in phosphate-buffered saline (PBS) containing dispase II (Dispase^®^, Roche, Mannheim, Germany) for 15 min to make the cells floating monolayer sheets. Released cell sheets were carefully transferred to 15 ml conical tubes with 5 ml PBS. The tubes were then subjected to mechanical stress by rotating 10–20 times in a tube rotator (Labquake™, Thermo Scientific, Waltham, MA). To give consistent and reproducible mechanical stress, a rotator was applied instead of the manual pipetting used in the literature [9]. PBS containing fragmented cell sheets was transferred to 35-mm culture dishes and then scanned by a digital scanner. The total number of fragments was counted by Image J software.

### Transmission electron microscopy (TEM)

Ultrastructures of desmosomes were analyzed using TEM images. After siRNA transfection, dispase II was treated as above mentioned to make floating monolayer sheets. Dispase was used to harvest the cells while maintaining the intercellular adhesive structure. Dispase primarily disrupts cell-extracellular matrix adhesion while leaving intercellular adhesion of epithelial cells intact [10]. The cell sheets were transferred to 1.5-ml microcentrifuge tubes with 1 ml PBS. Harvested sheets were centrifuged at 500 g for 3 min. After washing with PBS twice, the cell pellets were fixed overnight in a mixture of cold 2.5% glutaraldehyde and 2% paraformaldehyde in 0.1 M phosphate buffer (pH 7.2). The rest of the procedures were performed according to the common protocols for conventional electron microscopic exams (processed in the laboratory of the electron microscope, Seoul National University Hospital). More than 50 images were obtained per group, and the number of desmosomes used in size analysis was 193 and 207 in the control and *FUT1* knockdown groups, respectively.

### Immunoprecipitation

Primary keratinocytes were grown with KGM in the 100-mm dish until > 90% confluence, and the medium was switched to a high-calcium medium for an additional 48 h. Then cells were lysed in lysis buffer (1x RIPA) containing protease inhibitor cocktail (cOmplete™, Roche) and phosphatase inhibitor cocktail 2 (Sigma-Aldrich) on ice for 1 h with intermittent vortexing, and the supernatant was obtained by centrifugation at 13,000 rpm for 20 min was used for immunoprecipitation. For immunoprecipitation, 500 ug of cell lysates were incubated with 2 ug of mouse IgG or mouse monoclonal primary antibodies against H2 (BRIC231), and EGFR (A-10) and 30 ul of Protein A/G plus-Agarose (Santa Cruz Biotechnologies) for overnight at 4℃ on the rotator. Then, the samples were centrifuged at 3,000 rpm for 3 min, and the pellets were washed three times with 1x PBS at 3,000 rpm for 3 min. After the final wash, the pellets were added with 2x SDS sample buffer, and heated at 95℃ for 5 min. After cooling on ice, the samples were centrifuged at 13,000 rpm for 3 min, and the supernatants were subjected to the SDS-PAGE for Western blot analysis. All the procedures for Western blot analysis were the same as mentioned above, except using EasyBlot anti-mouse IgG (Genetex, Irvine, CA, USA) as an HRP-conjugated secondary antibody.

### Preparation of an adenovirus vector encoding human FUT1 gene

For overexpression of *FUT1* in primary human keratinocytes, we generated a recombinant adenovirus vector encoding human wild-type FUT1. FUT1 gene was amplified from human cDNA and subcloned into a pENTR(TM)/D-TOPO(TM) vector that has attL sites for site-specific recombination with a Gateway destination vector (Invitrogen, Carlsbad, CA). The replication-incompetent adenoviruses were created using a Vira-power adenovirus expression system (Invitrogen) in HEK293A cells, as previously described [11]. The amplified virus was purified by double cesium chloride-gradient ultracentrifugation. Viral particles in the cesium chloride gradient were collected and dialyzed using a Slide-A-Lyzer Dialysis Cassette (Thermo Scientific). An adenovirus vector encoding GFP was used as a control vector [11]. For adenovirus-mediated gene transduction, primary human epidermal keratinocytes were infected with an indicated multiplicity of infection (MOI) of the adenovirus in a high-calcium growth medium. If the incubation time is longer than 2 days, the culture medium was additionally changed with high calcium-containing fresh KGM at 48 h after infection.

### Real-time quantitative PCR

siRNA-transfected keratinocytes or adenoviral vector-treated keratinocytes were harvested at indicated time points for the analysis of mRNA expression, and total RNA was extracted using RNAiso plus (Takara Bio Inc., Shiga, Japan) according to the manufacturer’s protocols. 1 µg of total RNA was used to convert to cDNA using a First Strand cDNA Synthesis Kit (Thermo Fisher Scientific). Quantitative real-time PCR was performed on a CFX96™ Real-Time PCR Detection System (Bio-Rad Laboratories, Inc., Hercules, CA, USA) using 2X GreenStar™ qPCR Master Mix (Bioneer Corp., Daejeon, Korea) according to the manufacturer’s instructions. The PCR conditions were 95°C for 3 min, followed by 40 cycles at 95°C for 22 sec and 60°C for 1 min. The data were analyzed through the comparative *C*_T_ method (*ΔΔC*_T_ method), normalized to *36B4*, and presented as relative folds ± standard deviation. Primers used were: FUT1 forward: 5’-AAC AGA TCC GCA GAG AGT TCA, reverse: 5’-GCA TAA CCT GCA GAT AGT CCC, *AREG* forward: 5’-ACC TAC TCT GGG AAG CGT GA-3’ and reverse: 5’-AGC CAG GTA TTT GTG GTT CG-3’. *HBEGF* forward: 5′-CCT CCA GTG CTG GAT TTG AT-3′, and reverse: 5′-GCC AGG AAA TTG CCA AAG TA-3′, *TGFA* forward: 5′-CTT CAA GCC AGG TTT TCG AG-3′, and reverse: 5′- CTT CTG TGA CTG GGC AGG TT -3′, and *36B4* forward: 5’- TCG ACA ATG GCA GCA TCT AC -3’ and reverse: 5’- TGA TGC AAC AGT TGG GTA GC -3’.

### Enzyme-linked immunosorbent assay (ELISA)

Conditioned media were collected from keratinocytes at indicated time, and secretions of amphiregulin (AREG), heparin binding-epidemal growth factor (HB-EGF), and tumor growth factor-α (TGF-α) in conditioned media were measured by ELISA using commercial kits for human AREG (#CSB-04486 h), HB-EGF (#CSB-E09716h), and TGF-α (#CSB-E04724h), purchased from Cusabio Technology LLC, according to the manufacturer’s instructions. Data are represented as mean relative expression (%) ± SD relative to levels in the control groups (NC siRNA-transfected or control adenovirus-infected cells).

### Statistical analysis

Statistical analyses were performed using the Wilcoxon signed-rank test or one-way ANOVA followed by Bonferroni’s post-hoc test. Data are represented as the mean values ± SD of four or more independent experiments. Statistical significance was set at *p* < 0.05.

## Results

### The H antigen, a precursor to ABO blood type antigens, is present in the epidermis of normal human skin, along with the ABO antigen corresponding to each individual’s blood type

In the normal human epidermis, type 2 H antigen (H2 antigen) exhibits moderate expression in the mid and upper spinous layers, while being absent in the basal layer and lower spinous layers (see Additional file 1: Fig. S1). On the other hand, the corresponding A or B antigens, determined by individual blood type, are primarily expressed in the granular layers and horny layers of the epidermis (see Additional file 1: Fig. S1). Dermal blood vessel endothelial cells also express both H2 antigen and the corresponding A or B antigen. Despite their presence, the functional roles of H2 antigen and A/B blood group antigens in the human epidermis remain unclear, warranting further investigation to understand their potential contributions to skin physiology.

### *FUT1* knockdown increased cell-cell adhesive strength in the cultured primary human keratinocytes

**Fig. 1:**
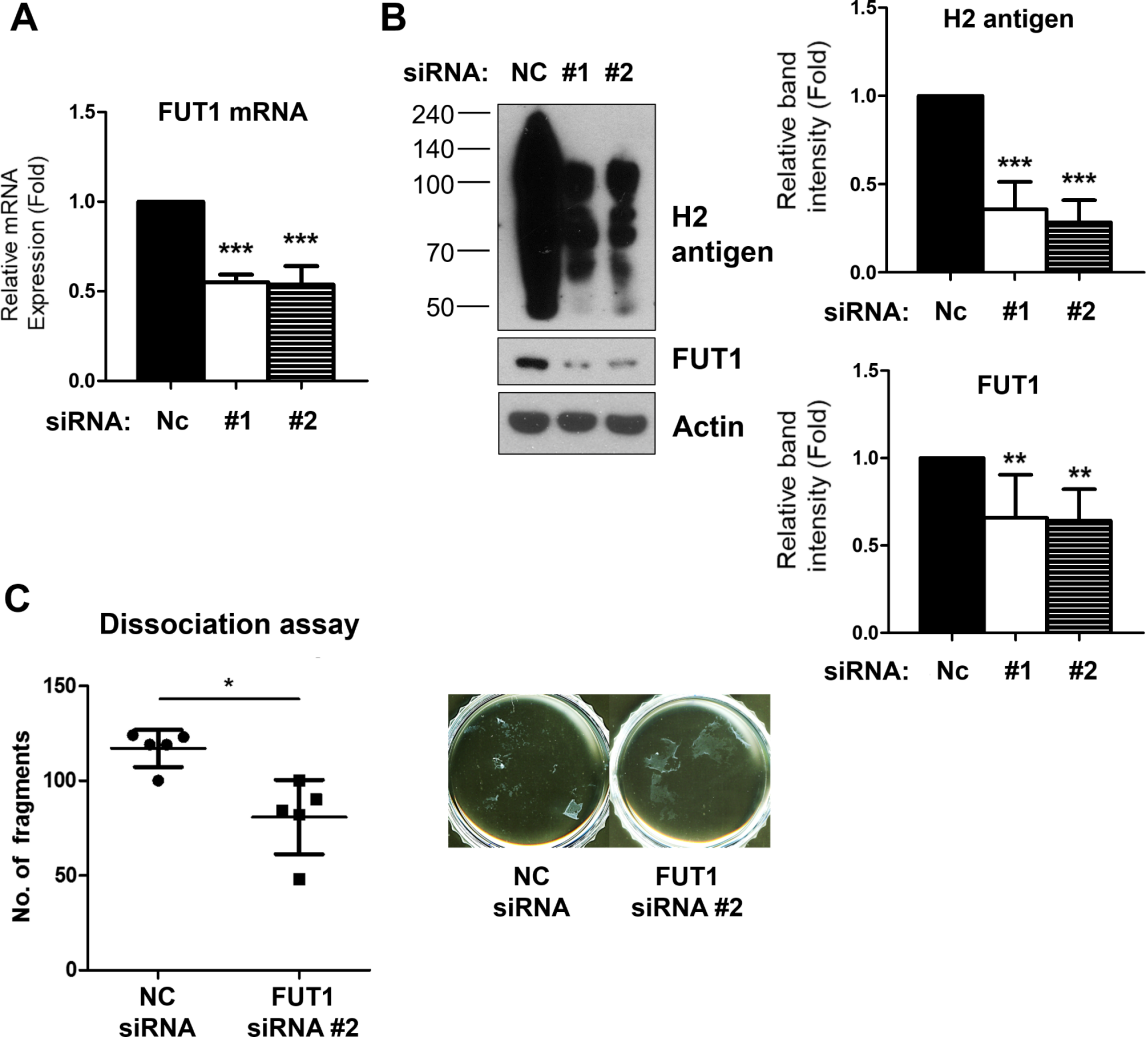
Downregulation of H2 antigen by *FUT1* knockdown enhances cell-cell adhesive strength in primary human keratinocytes. **A**,** B** Primary keratinocytes were transfected with negative control (NC) or *FUT1* (#1 or #2, two different pools of *FUT1* siRNA) siRNAs. **A** The downregulation of the FUT1 mRNA expression was measured using real-time qRT-PCR, normalized to *36B4* mRNA expression (*n* = 5). **B** The downregulation of the *FUT1* protein and its product, H2 antigen, were investigated through Western blot. β-actin was used as a loading control (*n* = 7). Relative mRNA expression or band intensity was presented as mean fold ± standard deviation (SD) to the NC siRNA-treated control group. **p < 0.01, ***p < 0.001 vs. NC siRNA-treated control group. **C** Primary keratinocytes were transfected with NC or *FUT1* (#2) siRNA cocktails. The cell-cell adhesive strength was measured by dispase-based dissociation assay. Data is depicted as a mean number of fragments ± SD (*n* = 5). *p < 0.05 vs. NC siRNA-treated control group

To explore the role of the H2 antigen, we initially investigated its impact on the cell-to-cell adhesions of epidermal keratinocytes. For knockdown of H2 antigen expression, we transfected a siRNA for α-(1,2)-fucosyltransferase 1 (FUT1) and assessed cell-cell adhesive strength using dispase-based dissociation assay, as described in the Methods. *FUT1* siRNA transfection significantly decreased FUT1 mRNA and protein expression, and robustly decreased H2 antigen expression in primary cultured keratinocytes (Fig. 1A, B), resulting in a notable reduction in the number of cell sheet fragments compared with control keratinocytes (Fig. 1C). This finding suggests that the H2 antigen, the precursor of the A/B blood group antigen, may play a crucial role in determining the strength of keratinocyte-keratinocyte adhesion.

### Increased desmosome size and elevated desmosomal protein levels induced by *FUT1* knockdown in cultured keratinocytes

**Fig. 2:**
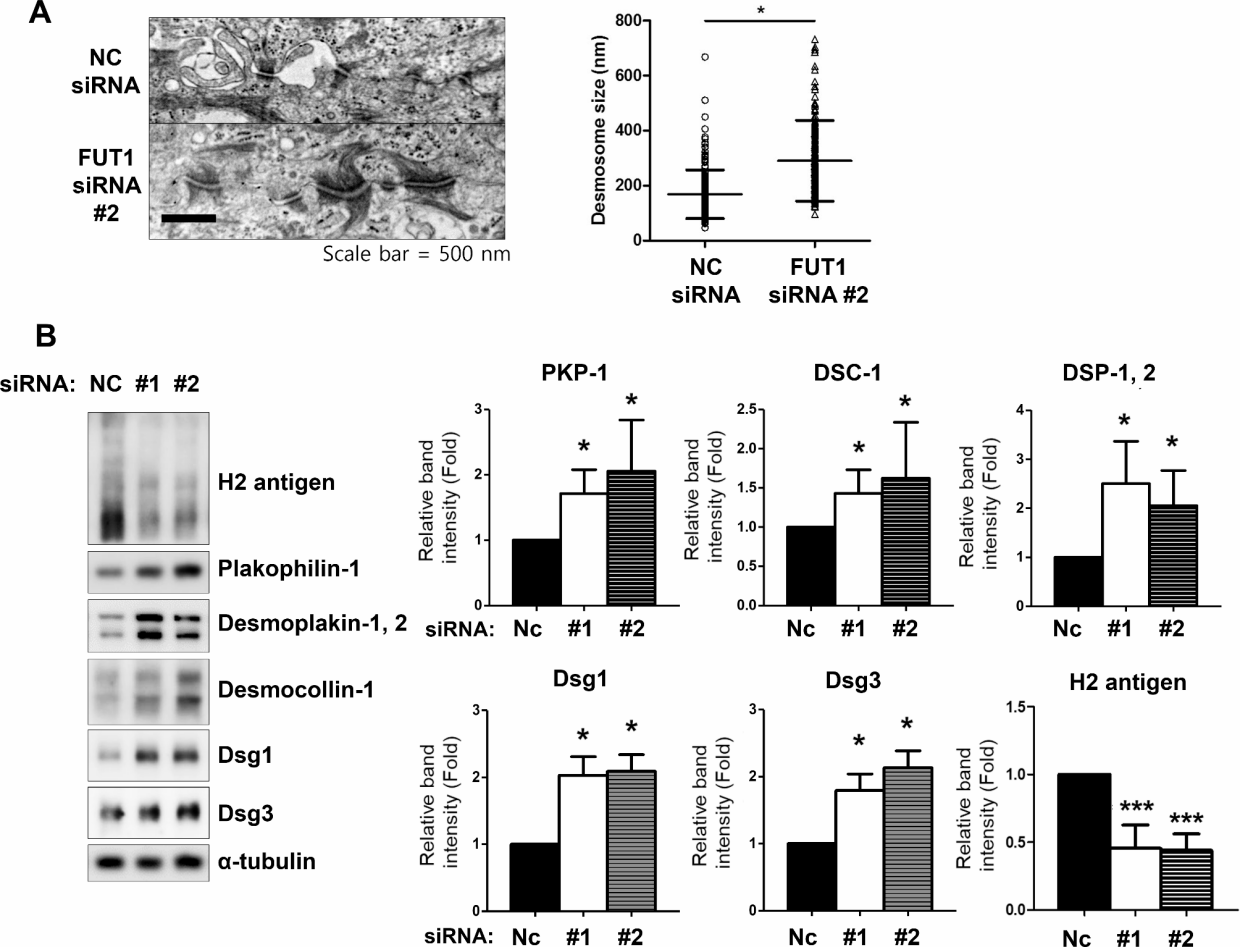
Downregulation of H2 antigen by *FUT1* knockdown increases desmosome size and expression of desmosomal proteins. **A** Primary keratinocytes were transfected with NC or *FUT1* (#2) siRNA cocktails. Cell sheets obtained from dispase treatment were processed for the transmission electron microscopic examination. More than 50 images per group were used to measure desmosome size using Image J software. Scale bar = 500 nm. Data were depicted as mean desmosome size (nm) ± SD. *p < 0.05, vs. NC siRNA-treated control group. **B** Primary keratinocytes were transfected with NC or *FUT1* (#1 or #2) siRNA cocktails. The levels of desmosome assembly proteins such as plakophilin, desmoplakin, desmocollin, and desmogleins with high calcium treatment (2 mM CaCl_2_) were investigated through Western blot. α-tubulin was used as a loading control. Relative band intensity was presented as mean fold ± SD to NC siRNA-treated control group. *p < 0.05, ***p < 0.001 vs. NC siRNA-treated control group

Given that the desmosome complex is a pivotal mediator of epidermal keratinocyte adhesion [2, 12–14], we investigated the desmosome ultrastructure in *FUT1* knockdown keratinocytes through transmission electron microscopy. While the overall desmosome structure remained unaltered, a striking increase in length was observed in siRNA-transfected cells (291 ± 147 nm compared with 169 ± 89 nm in control keratinocytes, *p < 0.05, Fig. 2A). This finding prompted us to explore the desmosomal protein expression. Consistent with the observed increase in desmosome size, *FUT1* knockdown significantly upregulated the expression of key desmosomal proteins such as desmogleins, plakophilin, desmocollin, and desmoplakin (Fig. 2B). Disturbed desmosomal adhesion contributes to the pathogenesis of several diseases such as pemphigus, which is caused by antibodies against desmosomal cadherins [15]. Our findings indicate that reduced FUT1 levels, leading to diminished H antigen expression, may contribute to forming larger and potentially stronger desmosomes through enhanced desmosomal protein production, leading to enhanced cell-cell adhesion.

### *FUT1* knockdown facilitated keratinocyte differentiation

**Fig. 3:**
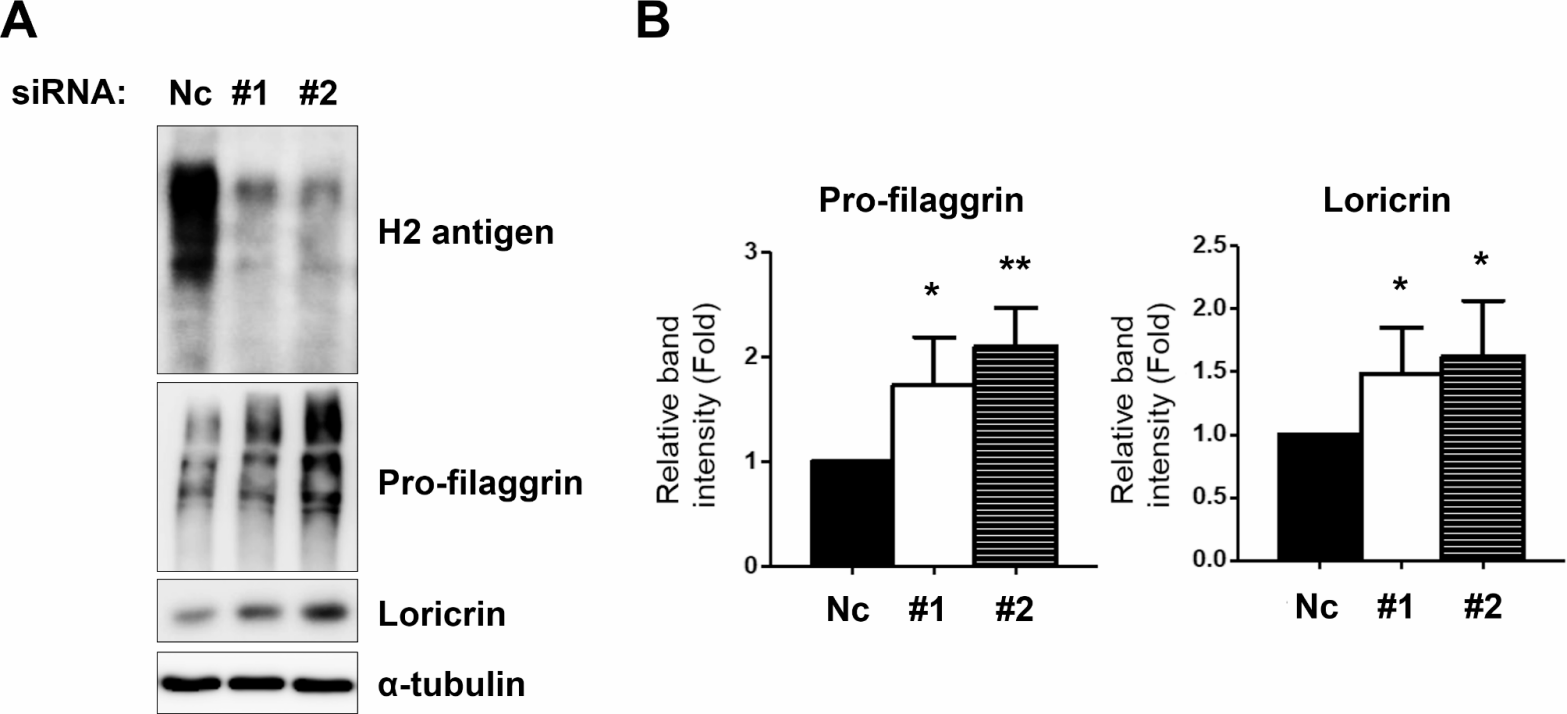
Downregulation of H2 antigen by *FUT1* siRNA transfection increases the expression of pro-filaggrin and loricrin. **A** Primary keratinocytes were transfected with NC or *FUT1* (#1 or #2) siRNA cocktails. The keratinocyte differentiation markers, including pro-filaggrin and loricrin, were investigated after four days of calcium treatment through Western blot (*n* = 5). α-tubulin was used as a loading control. **B** Relative band intensity was shown as mean fold ± SD to NC siRNA-treated control group. *p < 0.05, **p < 0.01 vs. NC siRNA-treated control group

In the previous literature, desmosome complex formation is associated with epidermal keratinocyte differentiation [16–18]. Therefore, we subsequently examine the keratinocyte differentiation upon reducing the H2 antigen. After 24 h of transfecting siRNA to the cultured keratinocytes, cells were incubated with a high calcium medium (2 mM) for 4 days. Downregulation of H2 antigen expression significantly increased the differentiation marker expressions such as pro-filaggrin and loricrin (Fig. 3A, B). These findings reveal that reducing H2 antigen by *FUT1* knockdown enhances keratinocyte differentiation.

### Inhibition of EGFR signaling recapitulated *FUT1* knockdown on keratinocytes

**Fig. 4:**
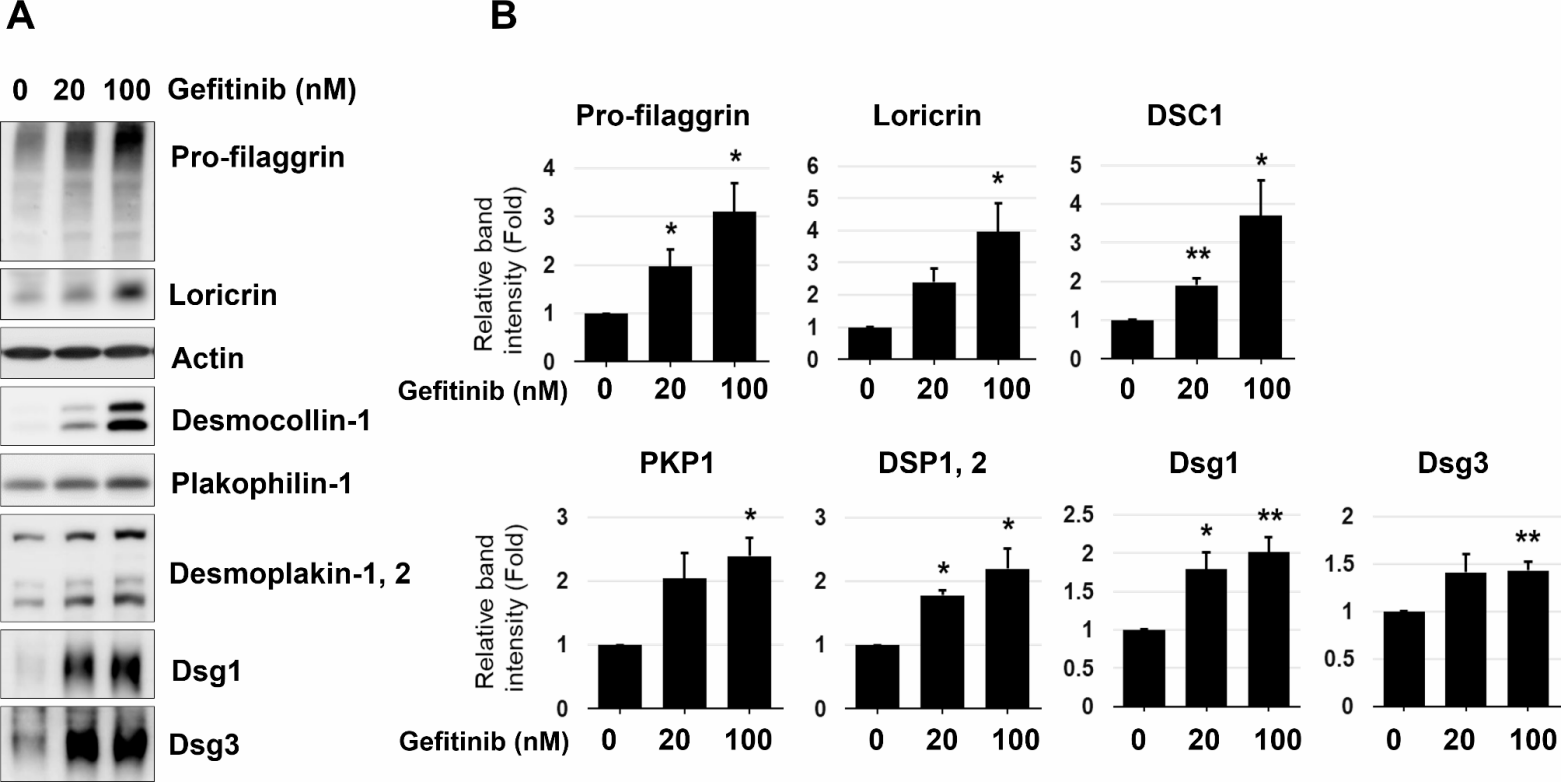
Treatment with an EGFR inhibitor, gefitinib, increases pro-filaggrin, loricrin, and desmosomal proteins. **A** Primary keratinocytes were treated with indicated doses of an EGFR inhibitor, gefitinib (0, 20, or 100nM). The level of proteins for keratinocyte differentiation markers and desmosome assembly following 4-day-calcium treatment were investigated through Western blot (*n* = 5 ∼ 6). β-actin was used as a loading control. **B** Relative band intensity was demonstrated as mean fold ± standard error of the mean (SEM) to NC siRNA-treated control group. *p < 0.05, **p < 0.01 vs. NC siRNA-treated control group

It is well known that desmosome assembly is regulated by EGFR signaling [18]. In addition, EGFR activation blocks the differentiation process of the keratinocyte [19]. Hence, to investigate the relationship between the EGFR signaling pathway and *FUT1* knockdown, primary human epidermal keratinocytes were treated with an EGFR inhibitor, gefitinib, with indicated doses of 0, 20, and 100 nM. The proteins for desmosomal assembly and the differentiation markers of keratinocytes were assessed upon treatment with gefitinib. Notably, the gefitinib treatment recapitulated the effects observed in *FUT1* knockdown experiments. Dose-dependent increases in desmosomal proteins and differentiation marker expressions were observed (Fig. 4A, B). These findings suggest that the reduction of H2 antigen by the *FUT1* knockdown affects the keratinocytes potentially through EGFR signaling.

### *FUT1* knockdown regulates the EGFR signaling via controlling the EGF ligand level

**Fig. 5:**
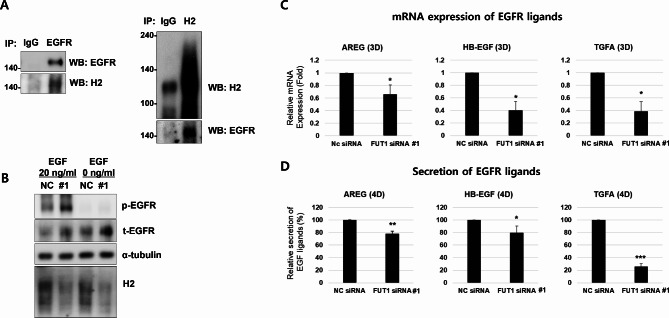
*FUT1* knockdown reduces EGFR ligands rather than directly regulating EGF receptors. **A** Primary keratinocytes were incubated with 2 mM CaCl_2_ for 2 days, and the cell lysates were harvested for immunoprecipitation (IP). IP was performed with antibodies against epidermal growth factor receptor (EGFR), H2 antigen, or mouse immunoglobulin (IgG). The glycosylation of H2 antigen on EGFR was confirmed through Western blot analysis.​ **B** Primary keratinocytes were transfected with NC or *FUT1* (#1) siRNA cocktails, and the phosphorylation of EGFR (Y1068) induced by treatment with EGF (0 or 20 ng/ml) was investigated through Western blot. EGF was treated in keratinocyte basal medium (KBM) for 5 min, with 24-hour pre-incubation in KBM before treatment. α-tubulin was used as a loading control. **C**,** D** Primary keratinocytes were transfected with NC or *FUT1* (#1) siRNA cocktails. The levels of mRNA expression **C** and protein secretion into conditioned medium **D** of EGFR ligand proteins following high calcium treatment for the indicated days were investigated using real-time qRT-PCR and ELISA (*n* = 5). The relative mRNA expression or secretion of EGFR ligands was presented as mean value (fold or %, respectively) ± SD to NC siRNA-treated control group. *p < 0.05, **p < 0.01, ***p < 0.001 vs. NC siRNA-treated control group

We investigated whether *FUT1* knockdown regulates EGFR signaling in primary human epidermal keratinocytes. EGFR is known to be ABH antigen-glycosylated and modulate EGF-EGFR binding activity [20, 21]. Therefore, we examined if this was the case in our in vitro setting. Our immunoprecipitation data validated H2 antigen-glycosylation of EGFR in primary cultured human keratinocytes (Fig. 5A). These findings led us to examine whether *FUT1* knockdown reduced EGFR signal. Treatment with one of the receptor ligands, epidermal growth factor (EGF), increased phosphorylated EGFR (p-EGFR) expression compared with the untreated group, as expected. However, the knockdown of the *FUT1* did not reduce the p-EGFR expression in a given ligand level compared with the NC control (Fig. 5B). Consequently, *FUT1* knockdown might indirectly reduce EGFR signaling rather than directly regulate EGFR activity. We then investigated the changes in endogenous EGFR ligand levels upon *FUT1* knockdown. EGFR ligand expressions at both mRNA and secreted protein levels were reduced upon *FUT1* knockdown (Fig. 5C, D). Overall, these results suggest that the decrease in H2 antigen-glycosylation via *FUT1* knockdown reduces EGFR signaling by lowering the EGFR ligand levels rather than regulating the response of EGFR itself in cultured human keratinocytes.

### *FUT1* overexpression showed a reversed effect via increased EGFR ligands

**Fig. 6:**
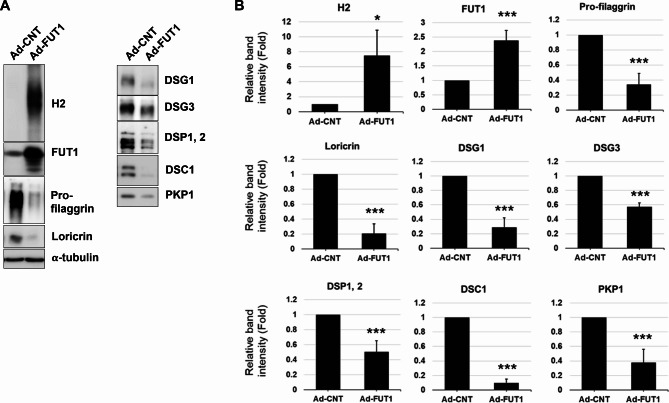
Enhanced expression of H2 antigen by *FUT1* overexpression reverses the effects shown in *FUT1* knockdown. **A** Primary keratinocytes were infected with 10 multiplicity of infection (MOI) of a control adenovirus vector encoding *GFP* gene (Ad-CNT) or recombinant adenovirus vector encoding human FUT1 gene (Ad-FUT1) in a high-calcium growth medium. The upregulation of the *FUT1* protein and its product, H2 antigen, were investigated through Western blot. Changes in proteins for keratinocyte differentiation and desmosome assembly were investigated four days after the infection through Western blot. α-tubulin was used as a loading control. **B** Relative band intensity was shown as mean fold ± SD to the Ad-CNT-infected control group. *p < 0.05, ***p < 0.001 vs. Ad-CNT-infected control group

**Fig. 7:**
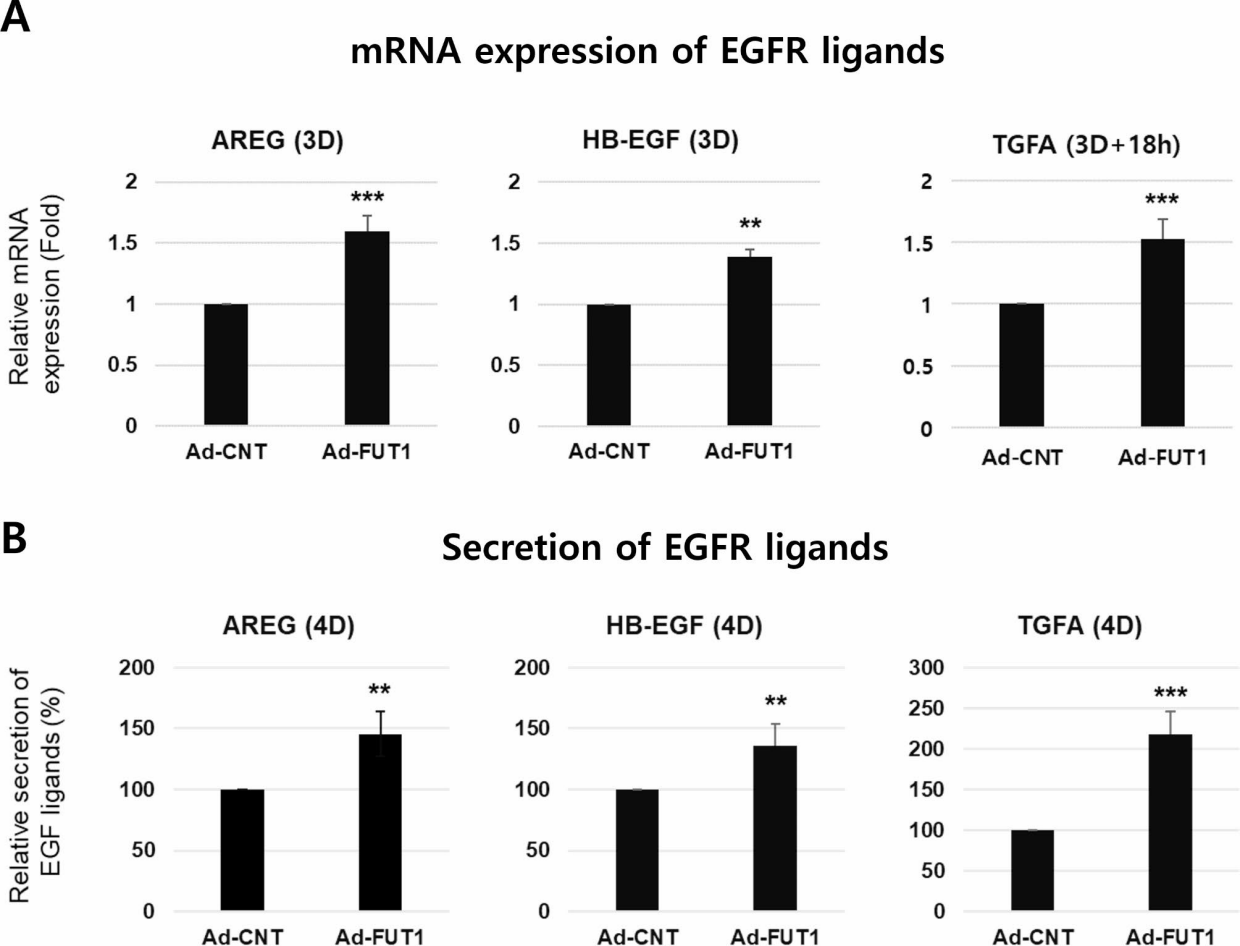
Overexpression of *FUT1* increases both mRNA and protein levels of EGFR ligands. Primary keratinocytes were infected with 10 MOI of a control adenovirus vector encoding *GFP* gene (Ad-CNT) or recombinant adenovirus vector encoding human FUT1 gene (Ad-FUT1) in a high-calcium growth medium. The levels of mRNA expression **A** and protein secretion into conditioned medium **B** of EGFR ligand proteins with high calcium treatment for the indicated days were investigated using real-time qRT-PCR and ELISA (*n* = 5). Relative mRNA expression or secretion of EGFR ligands was shown as mean value (fold or %, respectively) ± SD to the Ad-CNT-infected control group. **p < 0.01, ***p < 0.001 vs. Ad-CNT-infected control group

We further investigated whether *FUT1* overexpression in primary human epidermal keratinocytes showed reversed effects on desmosomal complexes and keratinocyte differentiation via altering EGFR ligand expressions. Recombinant adenovirus vectors encoding human FUT1 (Ad-FUT1) significantly upregulated H2 antigen and FUT1 expression compared with control vectors (Ad-CNT) (Fig. 6A; Additional file 1: Fig. S2). Notably, *FUT1* overexpression led to a significant downregulation of differentiation markers (Pro-filaggrin and Loricrin) and desmosomal proteins (desmogleins, desmoplakin, desmocollin, and plakophilin) compared with Ad-CNT-transfected cells (Fig. 6B). Further supporting this reversal, *FUT1* overexpression significantly increased both mRNA and secreted protein levels of EGFR ligands (Fig. 7A, B). Collectively, these findings suggest that elevated H2 antigen due to *FUT1* overexpression in primary human keratinocytes exerts opposite effects compared with *FUT1* knockdown. The observed downregulation of desmosomal proteins and differentiation markers likely stems from enhanced EGFR signaling driven by increased EGFR ligand expression.

## Discussion

In healthy human epidermis, H2 antigen is expressed in the upper suprabasal, granular, and horny layers, but not in the lower suprabasal or basal layer [3, 22]. Our study highlights the potential significance of the H2 antigen synthesized by FUT1 in keratinocyte cell-cell adhesion and differentiation. The results of this study revealed that the *FUT1* knockdown promoted desmosome assembly and increased the desmosome size, which led to improved cell-cell adhesive strength.

Glycosylation associated with FUT1, which synthesizes the H2 antigen or, additionally Lewis Y antigen depending on the cell type, can be observed across different epithelial cell lines. Studies have shown that FUT1 knockdowns are linked to decreased cell proliferation, tumorigenicity, and migration in several cancer cell lines or keratinocyte cell lines [6, 23–25], whereas overexpression of FUT1 enhanced tumorigenesis and migration in different cancer cell lines [24, 26]. These results might align with ours, where silencing FUT1 increased adhesion and decreased EGFR signaling. Furthermore, it has been shown that FUT1 is involved in a number of other cellular behaviors, including apoptosis [27], angiogenesis [28, 29], autophagy [30], and drug resistance [24] in different cell lines. However, it is unclear if it directly resulted from a reduced level of H antigen since cancer cells have various subtypes of Lewis antigen by further modifying H antigen. That is probably why reports indicate that FUT1 plays contradictory roles in adhesion, proliferation, and migration depending on the cell types [6–26, 31, 32].

Normal desquamation occurs when keratinocytes differentiate from the basal layer to the horny layer [33]. During the process, cell-cell adhesion should be properly controlled for optimal desquamation. It has been reported that desmosomes decreased in both sizes and numbers from the lower suprabasal layer to the upper suprabasal layer in normal skin [34]. Therefore, it is reasonable that the FUT1 is expressed in the upper epidermis and utilized to regulate desmosome morphology and quantity to control the cell-cell adhesive strength. Optimal desquamation is crucial to the skin barrier function in light of Netherton syndrome or psoriasis. Previous studies suggested that the horny layers of psoriasis and atopic dermatitis lesions contain hyperkeratotic scales, indicating a disturbed desquamation process [35, 36]. Psoriasis is a representative skin disease that harbors clinically evident silvery-white scales in the lesion [37]. Recently, our group observed decreased H2 antigen expression along with FUT1 level by immunofluorescence assay [5]. In the independent psoriasis and healthy cohort, we quantitatively demonstrated that the H2 antigen expression was reduced in psoriatic epidermis (see Additional file 1: Fig. S3). A recent study also reported that the expression of FUT1 and H2 antigen is reduced in the granular layers of the atopic dermatitis skin [38]. It remains unclear why this decrease in H2 antigen occurred, but we speculate that it may be associated with the clinical findings of the retained scale. Furthermore, in atopic dermatitis, corneodesmosomal proteins such as desmoglein 1 and desmocollin 1 exhibited elevated levels compared with normal or non-lesional skin, indicating increased cell-to-cell adhesion [36], which may have a relation to the regulation of H2 expression in atopic dermatitis skin.

Desmosome assembly requires a sufficient protein level of desmosomal cadherins (desmogleins) and clustering of desmosomal proteins to be an adhesive complex [39]. The desmosomal cadherins comprising desmogleins (Dsg)1–4 and desmocollins (Dsc)1–3 are connected to attachment plaques such as desmoplakin, which links the cadherins to the keratin cytoskeletons [15, 40, 41]. Desmoglein 1 not only maintains epidermal tissue integrity but also promotes keratinocyte differentiation [16]. Our findings showed that the *FUT1* knockdown upregulated both mRNA and protein expression levels of desmoglein 1 and 3, which indicated the potential promotion of desmosomal assembly and keratinocyte differentiation. In addition, *FUT1* knockdown increased plakophilin 1 expression. It has been reported that plakophilin 1 enhanced the clustering of desmosomal proteins into desmosomes, promoting desmosome formation. When plakophilin 1 was overexpressed in primary human keratinocytes, desmosome formation occurred even in low calcium concentrations, and the desmosome size increased [42]. Moreover, cell migration was inhibited in *FUT1* knockdown HaCaT cells, the spontaneously immortalized cell lines from epidermal keratinocytes [6]. This is in line with the results in the present study that *FUT1* knockdown enhanced desmosome formation. For keratinocyte migration, desmosome remodeling and disassembly should occur [43]. It is sensible that the increased cell-to-cell adhesion may hinder cell migration.

The keratinocyte differentiation process creates the epidermal barrier, which is vital to the survival of mammals. Signal transduction and gene expression pathways essential in controlling keratinocyte differentiation are known to be regulated by EGFR [19]. EGFR activation abrogates all known fundamental stages of keratinocyte differentiation while promoting keratinocyte proliferation and migration. The EGFR in A431 cells, the squamous cell carcinoma cell lines, are glycosylated by A antigen, as well as Lewis Y antigen, another product of FUT1 that is mostly expressed in cancer cells, and these sugar structures influence the binding of the EGF to its binding site on the receptor [20, 25–44]. This finding indicates that FUT1-mediated fucosylation of EGFR may regulate the receptor function. Other studies demonstrated that the knockdown of *FUT1* reduces EGFR signaling, probably mediated by downregulation of A and/or Lewis Y antigen, making the cancer cells more sensitive to EGFR deprivation [45, 46]. Our study also showed that in human primary keratinocytes, knockdown of *FUT1* demonstrated decreased EGFR signaling, which was recapitulated by the EGFR inhibitor. Thus, FUT1 expression may regulate desmoglein expression via the EGFR pathway.

To see if FUT1 regulates desmosomal proteins via directly modifying their glycosylation, we tried immunoprecipitation through either an anti-desmogelin1/3 antibody or an H2 glycosylation antibody. Our experimental condition failed to detect H2 glycosylation on desmogleins (data not shown). Although we successfully detected H2 glycosylation on EGFR protein, the receptor response to the fixed dose of EGF was not decreased by *FUT1* knockdown. This may be due to lack of Lewis Y antigen expression in epidermal keratinocytes and suggests that Lewis Y may be involved in the direct binding or response of EGFR to the ligands, while H2 antigen may affect EGFR signaling indirectly. Our findings also revealed that promoting enhanced FUT1-induced fucosylation in human epidermal keratinocytes downregulated the expressions of desmosomal proteins and differentiation markers that potentially stemmed from increased EGFR signaling via EGF ligand expression.

## Conclusion

Altered expression of FUT1 in the epidermis possibly modulates cell-cell adhesion and keratinocyte differentiation status through regulation of H2 antigen and EGFR ligand expression, at least in part, which may eventually affect skin barrier function. Thus, our findings indicate a role for H2 antigen glycosylation in keratinocytes, mediated by FUT1, in controlling the state of the epidermal barrier.

## Electronic supplementary material

Below is the link to the electronic supplementary material.

**Additional File 1**: **Figure S1**. Immunohistochemical staining of A, B, or H2 antigen in human skin. The formalin-fixed paraffin-embedded healthy human skin tissues were stained with antibodies against A antigen (Z2B-1, mouse IgM, 1:100), B antigen (Z5H-2, mouse IgM, 1:100), or H2 antigen (BRIC231, mouse IgG, 1:100) from Santa Cruz Biotechnology. The protocol for obtaining healthy skin from healthy volunteers using a punch biopsy method was approved by the medical ethics committee of the Institutional Review Board of Seoul National University Hospital (IRB No. C-1312-084-543), and all participants provided written informed consent. The study was conducted in accordance with the principles described in the Declaration of Helsinki. Immunohistochemical staining of H2 antigen presented at upper spinous, granular, and horny layers (A), and A or B antigen exhibits at granular layers and horny layers in the epidermis (B). **Figure S2.** Dose-dependent enhanced expression of H2 antigen by overexpression of *FUT1* in primary human epidermal keratinocytes. Primary keratinocytes were infected with 0, 2.5, 5, or 10 multiplicity of infection (MOI) of a control adenovirus vector encoding GFP gene (Ad-CNT) or recombinant adenovirus vector encoding human FUT1 gene (Ad-FUT1) in a high-calcium growth medium. The upregulation of the FUT1 protein and its product, H2 antigen, were investigated through Western blot at 2 days after infection for the confirmation of overexpression of FUT1 protein and its activity. α-tubulin was used as a loading control. **Figure S3**. Reduced H2 antigen expression in psoriatic epidermis. The left panel shows the representative images of immunofluorescence analysis in healthy controls and in the lesional skin of patients with psoriasis (H2 antigen (green) and DAPI (blue)). The right panel presents the quantitative analysis of H2 expression at upper spinous, granular, and horny layers. The graph depicts the relative intensity of H2 antigen staining per area in the healthy and psoriatic epidermis as mean percentages ± SEM with six volunteers in each group. **P < 0.01 by Mann-Whitney U test

## Data Availability

All relevant data are available within the article and its supplementary information files or from the corresponding authors upon reasonable request.

## Abbreviations

FUT1: Fucosyltransferase1
KGM: Keratinocyte growth medium
PBS: Phosphate-buffered saline
TEM: Transmission electron microscopy
MOI: Multiplicity of infection
ELISA: Enzyme-Linked Immunosorbent Assay
EGFR: Epidermal growth factor receptor
EGF: Epidermal growth factor
p-EGFR: Phosphorylated epidermal growth factor receptor
Dsg1: Desmoglein 1
Dsg3: Desmoglein 3
PKP1: Plakophilin 1
DSC1: Desmocollin 1
DSP1, 2: Desmoplakin 1, 2
t-EGFR: Total epidermal growth factor receptor
AREG: Amphiregulin
HB-EGF: Heparin-binding epidermal growth factor
TGFA: Tumor growth factor α
