# Supplementary material for: H Antigen expression modulates epidermal Keratinocyte Integrity and differentiation
Source: Biol Res. 2024 Oct 18;57:72. doi: 10.1186/s40659-024-00541-x (PMC11487879; doi:10.1186/s40659-024-00541-x)
Supplement: Supplementary file 1 — Additional File 1: Figure S1. Immunohistochemical staining of A, B, or H2 antigen in human skin. The formalin-fixed paraffin-embedded healthy human skin tissues were stained with antibodies against A antigen (Z2B-1, mouse IgM, 1:100), B antigen (Z5H-2, mouse IgM, 1:100), or H2 antigen (BRIC231, mouse IgG, 1:100) from Santa Cruz Biotechnology. The protocol for obtaining healthy skin from healthy volunteers using a punch biopsy method was approved by the medical ethics committee of the Institutional Review Board of Seoul National University Hospital (IRB No. C-1312-084-543), and all participants provided written informed consent. The study was conducted in accordance with the principles described in the Declaration of Helsinki. Immunohistochemical staining of H2 antigen presented at upper spinous, granular, and horny layers (A), and A or B antigen exhibits at granular layers and horny layers in the epidermis (B). Figure S2. Dose-dependent enhanced expression of H2 antigen by overexpression of FUT1 in primary human epidermal keratinocytes. Primary keratinocytes were infected with 0, 2.5, 5, or 10 multiplicity of infection (MOI) of a control adenovirus vector encoding GFP gene (Ad-CNT) or recombinant adenovirus vector encoding human FUT1 gene (Ad-FUT1) in a high-calcium growth medium. The upregulation of the FUT1 protein and its product, H2 antigen, were investigated through Western blot at 2 days after infection for the confirmation of overexpression of FUT1 protein and its activity. α-tubulin was used as a loading control. Figure S3. Reduced H2 antigen expression in psoriatic epidermis. The left panel shows the representative images of immunofluorescence analysis in healthy controls and in the lesional skin of patients with psoriasis (H2 antigen (green) and DAPI (blue)). The right panel presents the quantitative analysis of H2 expression at upper spinous, granular, and horny layers. The graph depicts the relative intensity of H2 antigen staining per area in the health [file 40659_2024_541_MOESM1_ESM.pdf]

Additional File 1 for *H Antigen Expression Modulates Epidermal Keratinocyte Integrity and Differentiation*

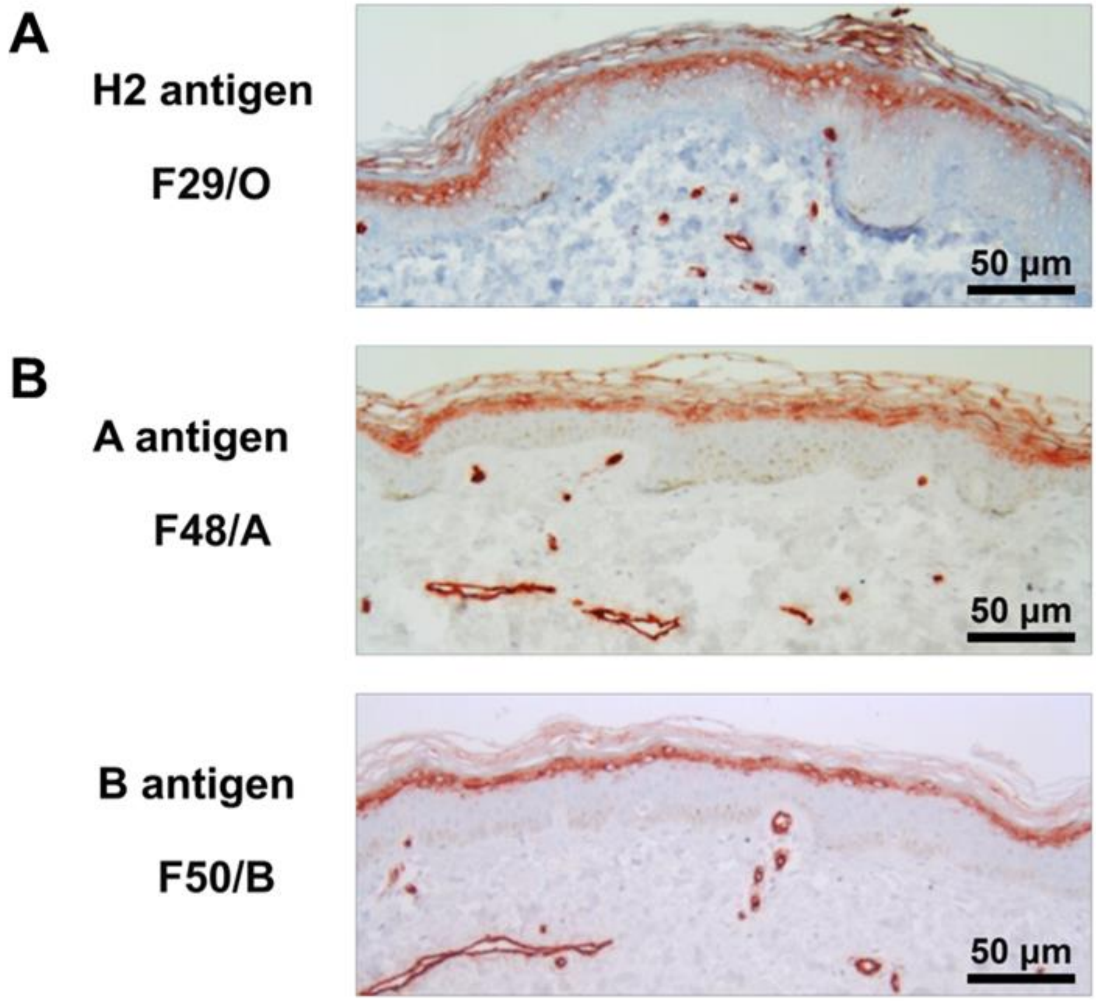

Figure S1. Immunohistochemical staining of A, B, or H2 antigen in human skin.

The formalin-fixed paraffin-embedded healthy human skin tissues were stained with antibodies against A antigen (Z2B-1, mouse IgM, 1:100), B antigen (Z5H-2, mouse IgM, 1:100), or H2 antigen (BRIC231, mouse IgG, 1:100) from Santa Cruz Biotechnology. The protocol for obtaining healthy skin from healthy volunteers using a punch biopsy method was approved by the medical ethics committee of the Institutional Review Board of Seoul National University Hospital (IRB No. C-1312-084-543), and all participants provided written informed consent. The study was conducted in accordance with the principles described in the Declaration of Helsinki. Immunohistochemical staining of H2 antigen presented at upper spinous, granular, and horny layers **(A)**, and A or B antigen exhibits at granular layers and horny layers in the epidermis **(B)**.

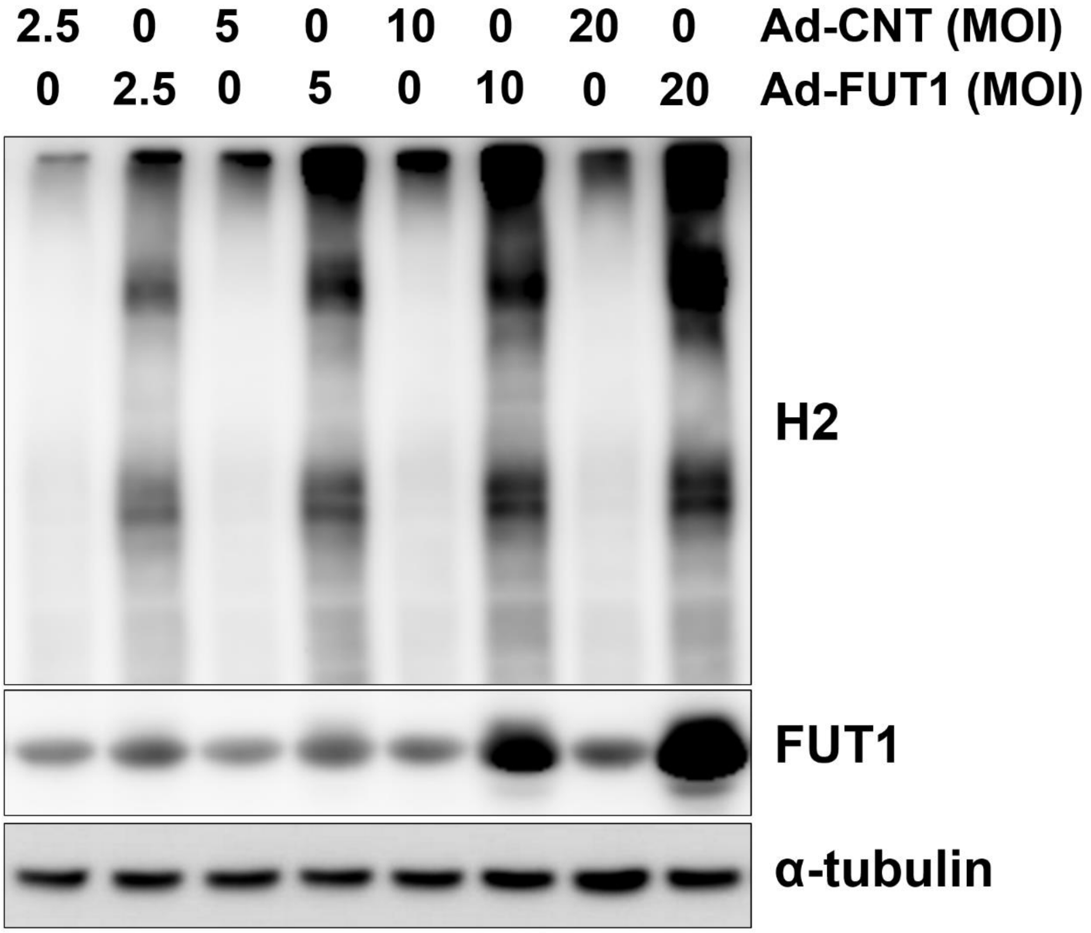

Figure S2. Dose-dependent enhanced expression of H2 antigen by overexpression of FUT1 in primary human epidermal keratinocytes.

Primary keratinocytes were infected with 0, 2.5, 5, or 10 multiplicity of infection (MOI) of a control adenovirus vector encoding GFP gene (Ad-CNT) or recombinant adenovirus vector encoding human FUT1 gene (Ad-FUT1) in a high-calcium growth medium. The upregulation of the FUT1 protein and its product, H2 antigen, were investigated through Western blot at 2 days after infection for the confirmation of overexpression of FUT1 protein and its activity. α-tubulin was used as a loading control.

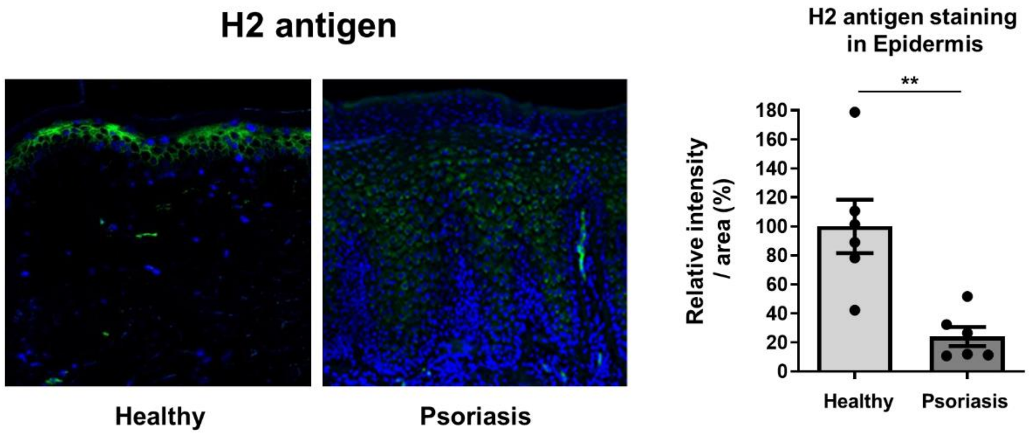

Figure S3. Reduced H2 antigen expression in psoriatic epidermis.

The left panel shows the representative images of immunofluorescence analysis in healthy controls and in the lesional skin of patients with psoriasis (H2 antigen (green) and DAPI (blue)). The right panel presents the quantitative analysis of H2 expression at upper spinous, granular, and horny layers. The graph depicts the relative intensity of H2 antigen staining per area in the healthy and psoriatic epidermis as mean percentages ± SEM with six volunteers in each group. $^{**}P < 0.01$ by Mann-Whitney U test.
